# Cervical Tuberculous Lymphadenitis: Clinico-demographic Profiles of Patients in a Secondary Level Hospital of Bangladesh

**DOI:** 10.12669/pjms.323.9550

**Published:** 2016

**Authors:** Mohammad Shah Kamal, Md. Hafiz Ehsanul Hoque, Fazle Rabbi Chowdhury, Rubina Farzana

**Affiliations:** 1 Mohammad Shah Kamal, FCPS (ENT). Junior Consultant (ENT), Shaheed Shamsuddin Ahmed Hospital, Sylhet, Bangladesh; 2 Md. Hafiz Ehsanul Hoque, PhD. MAG Osmani Medical College& Hospital, Sylhet, Bangladesh; 3 Fazle Rabbi Chowdhury, FCPS (Medicine). MAG Osmani Medical College& Hospital, Sylhet, Bangladesh; 4 Rubina Farzana, MBBS. MAG Osmani Medical College& Hospital, Sylhet, Bangladesh

**Keywords:** Tuberculosis, Mycobacterial Cervical lymphadenitis, Bangladesh

## Abstract

**Objective:**

Tuberculosis (TB) is a major public health problem in Bangladesh since long. The present incidence and prevalence rates of all forms of TB are 227 and 404/100,000 population respectively. The aim of this study was to find out the clinical characteristics of involved cervical lymph nodes, demographic characteristics of the patients and response to treatment of Cervical Tuberculous Lymphadenitis (CTL) cases.

**Methods:**

A prospective study was performed in Shaheed Shamsuddin Ahmed Hospital, Sylhet, Bangladesh from June 2012 to June 2014. Total 65 patients having CTL attending outpatient department of the hospital were enrolled.

**Results:**

Age of the patients ranged from 5 to 60 years with a mean of 25.6 years. Two third (67.7%) of the patients were female. Male: Female ratio was 1:2.1. More than half of the patients came from rural areas (53.8%) and from low socio-economic conditions (58.5%). Most of the patients presented with unilateral (87.7%), multiple (82.3%), matted (68.6%) lymph nodes, <3cm diameter (54%), commonly in right side (57.9%). Abscess was found in 21.5% cases. Discharging sinus was found in 9.2% cases. Most commonly involved lymph node group was level V (59.4%) followed by level II (42.2%). Systemic features were found in 63.07% patients. Associated lung lesion was found in 3.1% cases. FNAC was found positive for tuberculosis in 83.9% cases. Most of the patients (78.46%) were cured with six months anti-tubercular chemotherapy.

**Conclusions:**

Early diagnosis and treatment is critical in reducing the overall prevalence. It is essential to have awareness regarding common presentations of cervical tuberculous lymphadenitis among the general population as well as healthcare professionals working in the resource poor primary and secondary level hospitals.

## INTRODUCTION

Tuberculosis (TB) is a major public health problem in Bangladesh since long. In 2014, the incidence of all forms of TB for all age groups was 227/100,000 population, while the prevalence rate was 404/100,000 population. Globally in 2014, TB killed 1.5 million of people and 9.6 million of people estimated to have fallen ill with TB. TB now ranks alongside HIV as a leading cause of death worldwide.^1^

Extra-pulmonary TB constitutes 15-20% of all cases of Tuberculosis.^2^ TB Lymphadenitis is seen in nearly 35% of extra-pulmonary cases.^2^ Cervical lymph nodes are the most common site of involvement (60-90%).^2^ Tuberculous lymphadenitis historically named ‘scrofula’. The word ‘scrofula’ comes from the Latin *scrofulae* meaning brood sow. In the Middle ages, it was believed that the ‘royal touch’ of the sovereign of England or France could cure the disease. It was therefore known as the King’s evil.^3^ Cervical Tubercular Lymphadenitis (CTL) remains both diagnostic and therapeutic challenge because it mimics other pathologic process and yields inconsistent physical and laboratory finding.^4^ There is little information regarding CTL in Bangladesh. This study will help the healthcare professionals in diagnosis and treatment of CTL, especially those working in the primary and secondary level hospitals of Bangladesh.

## METHODS

### Study site and objectives

A prospective study was performed in Shaheed Shamsuddin Ahmed Hospital, Sylhet, Bangladesh, from June 2012 to June 2014. We selected 65 patients having CTL attending outpatient department of the hospital.

### Diagnostic tools

After a detailed history and clinical examination, FNAC was performed in all patients. Excision biopsy was performed when FNAC was negative or doubtful and clinical suspicion was high for tuberculosis. Abscess was diagnosed by incision and drainage of abscess with taking biopsy from abscess wall. Routine investigations including ESR, Chest Radiograph was done in all the patients. HIV screening was not performed in any of the patients as there was no clinical suspicion regarding AIDS.

### Demographic variables

For describing socio-economic status, we arbitrarily divided the patients into low (monthly household income <10000 taka), lower-middle (10000-20000 taka) and middle (>20000 taka). The age group was also subdivided in to four groups; less than 15, 16-30, 31-45 and 46- 60 years.

### Lymph node characteristics

For describing lymph nodes in the neck we used the system which originates from Memorial Sloan- Kettering Hospital, New York and adopted by the American Academy of Otolaryngology Head and Neck Surgery: Level I, Sub-mental and Sub-mandibular lymph nodes; Level II, Cervical jugular chain nodes above the level of hyoid; Level III, Cervical jugular chain nodes from the level of hyoid to the level of Cricoid; Level IV, Cervical jugular chain nodes from the level of Cricoid to the supra-sternal notch; Level V, Posterior triangle lymph nodes; Level VI, Central compartment nodes.^5^

### Mode of treatment

After confirming diagnosis of CTL all patients were treated according to national guidelines: Category one for new cases (first two months isoniazid, rifampicin, pyrazinamide and ethambutol followed by isoniazid and rifampicin for next four months), Category II for default cases (first three months isoniazid, rifampicin, pyrazinamide and ethambutol followed by isoniazid and rifampicin for next five months). Along with medical treatment, surgical treatment was given in the form of excision of the large lymph nodes (>6cm) suspected not to respond by medical treatment only, incision and drainage of abscess and excision of sinus tract along with associated lymph nodes. All patients were followed up at least six months to one year (2 monthly for six months than 3 monthly for next six months) and progress was assessed by clinical examination. In this study we defined ‘cure’ as complete disappearance of lymph nodes or decrease in size of <1cm.

### Statistical analysis

Data were analyzed by Statistical Package for Social Sciences [SPSS version 20]. They were presented as table with percentage and proportion. Bivariate analysis with gender was done in some of the variables. Chi-square and fisher’s exact test was done. P value was used to show the level of significance.

## RESULTS

Total 65 patients of CTL were included in this study. The age of the patients ranged from 5 to 60 years with a mean age of 25.6 years (SD=13.26). The commonest age group was 16-30 years (61.5%). Two third (67.7%) of the patients were female. Male:Female ratio was 1:2.1. Female patients were mostly housewives (56.8%). The other occupations include student 24.6%(16), day labor 10.8%(7), business 9.2%(6), house maid 6.2%(4) and unemployed 10.8%(7). More than half of the patients came from rural areas (53.8%). Socio-economic condition was low in 58.5% and lower-middle in 36.9% patients. Only 4.6% patients came from middle class family. Previous history of TB exposure was found in 26.2% cases (Table-I). 16-30 years age group were varied significantly in respect to gender though other socio-economic variables were not.

**Table-I:** Socio-demographic characteristics of the patients.

*Characteristics*	*Number (%)*	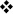 *P value*
*Sex (n=65)*		0.437
Male	21(32.3%)	
Female	44 (67.7%)	
*Age(in years)*		0.05
<15	10(15.3%)	
16-30	40 (61.5%)	
31-45	8 (12.3%)	
46-60	7 (10.7%)	
*Residence of the patients (n=65)*		0.379
Rural	35(53.8%)	
Semi-urban	8(12.3%)	
Urban	22(38.3%)	
*Socio-economic condition (n=65)*		0.322
Low	38(58.5%)	
Lower-middle	24(36.9%)	
Middle	3(4.6%)	
*Occupation(n=65)*		
House wife	25(38.5%)	
Student	16(24.6%)	
Day labour	7(10.8%)	
Business	6(9.2%)	
House maid	4(6.2%)	
Unemployed	7(10.8%)	
*History of TB exposure (n=65)*		0.759
Yes	17(26.2%)	
No	48(73.8%)	

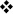
 Chi square and Fisher’s exact test was applied

Among the 65 patients, 45 (69.2%) presented with solid lymph nodes, 14 (21.5%) with abscess and 6 (9.2%) with discharging sinus. Unilateral involvement was found in 87.7% cases. Right side represented more (57.9%; 33) than left side (42.1%; 24). Multiple lymph nodes were found in 82.3% cases. Of them 68.6% (35) were matted and 31.4% (16) were discrete. Size of the largest lymph node was<3cm diameter in 54.8%, 3-6 cm in 37.1% and >6cm in 8.1% cases. Commonly involved lymph node group was level V (59.4%) followed by level II (42.2%) and level III (18.8%). Systemic features were found in 41 (63.07%) cases. Fever (87.8%; 36), Weight loss (63.4%; 26), Night sweat (63.4%; 26) and Loss of appetite (80.5%; 33) were the common systemic features (Table-II). Site of involvement and number of enlarged lymph nodes were not varied significantly in respect to gender of the patients.

**Table-II:** Clinical characteristics of involved Lymph nodes.

*Characteristics*	*Number (%)*	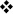 *P value*
*Mode of Presentation(n=65)*		0.103
Solid nodes	45(69.2%)	
Abscess	14(21.5%)	
Discharging sinus	6(9.2%)	
*Number of enlarged lymph nodes (n=62)*		0.276
Single	11(17.7%)	
Multiple	51(82.3%)	
Matted	35(68.6%)	
Discrete	16(31.4%)	
*Site of involvement(n=65)*		0.201
Unilateral	57(87.7%)	
Right	33(57.9%)	
Left	24(42.1%)	
Bilateral	8(12.3%)	
*Size of involved nodes(n=62)*		0.327
<3 cm	34(54.8%)	
3-6 cm	23(37.1%)	
>6 cm	5(8.1%)	
Level of involvement(n=65)*		
Level I	6(9.4%)	
Level II	27(42.2%)	
Level III	12(18.8%)	
Level IV	9(14.1%)	
Level V	38(59.4%)	
Systemic features(n=41)*		
Fever	36(87.8%)	
Weight loss	26(63.4%)	
Night sweat	26(63.4%)	
Loss of appetite	33(80.5%)	

* Multivariate response,

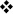
 Chi square and Fisher’s exact test was applied

FNAC was done in 62 cases and found significantly (p= < 0.01) positive in 52 (83.9%) cases. Chest radiograph was done in all cases and was normal (p= <0.01) in 96.9% (63) cases. ESR was raised (>30mm/1^st^ hour) in 70.76% cases with a limit of 5-87 and mean was 39.28mm/1^st^ hour (SD=23.21). Category I treatment was given in 60 cases. Most of the cases (85%, 51) cured within 6 months, few (10%, 6) needed 9 months and very few (5%, 3) needed one year treatment for their delayed response. Category II treatment was given in five (7.6%) cases. Surgical intervention was needed in 33 (50.8%) cases. No case was found ‘resistant’ or ‘relapse’ during the period of 6-12 months follow-up (Table-III). No significant relationship was found between the type of treatment and gender of the patients.

**Table-III:** Laboratory investigations and treatment of the patients.

*Characteristics*	*Number (%)*	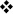 *P value*
*FNAC (n=62)*		<0.01
Positive for TB	52(83.9%)	
Negative for TB	10(16.1%)	
*Chest radiography (n=65)*		<0.01
Negative for TB	63(96.9%)	
Positive for TB	2(3.1%)	
*ESR(mm/1st hour)(n=65)*		0.378
Highest	87	
Lowest	5	
Mean	39.28	
*Treatment(n=65)*		
Medical treatment only	32(49.2%)	
Medical & surgical treatment	33(50.8%)	

## DISCUSSION

Robert Koch discovered the tubercular bacilli on 24 March 1882.^6^ Today, tuberculosis is still a major public health problem worldwide. Tuberculosis is more prevalent in low income countries like Bangladesh, India and Pakistan, as compared to high income countries.^7^ Globally in 2014, the number of TB death averaged 16 / 100000 population and 21 when TB death among the HIV–positive people included.^1^ It was more than 40 in five high–burden countries such as Afghanistan, Bangladesh, Cambodia, Indonesia and Myanmar.^1^ In the United States, there was a decline in the disease until 1980 but a resurgence thereafter, seeming to coincide with an increase in HIV infections. Since this time, tuberculosis has become a new clinical entity due to its changing patterns of clinical presentation and dissemination, with increasing prevalence of extra-pulmonary forms.^8^

Sex distribution of the patients (male:female;1:2.1) in this study was quite consistent with the studies done by Ahmed I *et al* in Pakistan(male 16.6%, female 83.4% with male: female ratio 1:5) and Jha BC *et al* in India (male 42.85%, female 57.15% with Male:Female ratio 1:1.3), but inconsistencies were found with the studies done by Magsi PB *et al* in Pakistan (male 57.14%, female 42.86% with Male:Female ratio 1.33:1) and Choudhury N *et al* in UK (male 63.63%, female 36.36% with Male:Female ratio 1.75:1).^9^-^12^ In this study female patients were found in much higher percentage than male and it may be due to social dynamics of the country. The majority of the female tends to stay or work inside their house in closed environment. So, less air ventilation tends to increase the overall risk of developing infectious diseases. In this study significant number of patients belonged to 16-30 years age group. Jha BC *et al* found the age of the patients ranged from 9 months to 62 years with a mean age of 23.7 years and commonest age group affected was 11-20 years (41.07%).^10^ The finding was quite consistent with our study. But in another study done by Choudhury N *et al*, revealed the age of the patients ranged from 21 years to 75 years with a mean age of 40 years.^12^

Demographic profiles of this study were similar with a Bangladeshi study (66.2%) and some other regional studies (in India 62.5% and in Pakistan 70%)where majority of the patients belonged to rural areas and from lower socio-economic group.^10^,^11^,^13^ Jha BC et al found the number of patients having cervical abscess or sinus quite low (5% each) though this study found much higher in percentage.^10^ Mogre DA in India examined 104 cases of tuberculous lymphadenitis and found no case with abscess or sinus formation.^14^ Abscess and sinus formation were found much higher in this study compared to others possibly because of delayed presentation as most of the patients came from rural areas and from low income groups. This study found unilateral neck swelling as the commonest (87.7%) involvement which was similar to the study done by Frontanilla JM et al (85%) but much higher than Jha BC et al (67.8%).^10^,^15^ Jha BC et al found multiple lymph nodes in 57% cases, of which matted 71.9% and discrete 28.1% which were quite consistent with our study.^10^ In the contrary, Mogre DA found single, mobile lymph node 81.80% and only 0.69% lymph nodes showed matting.^14^ The findings of involved lymph node groups in this study were consistent with the studies done by Magsi PB et al (level V followed by level I) and Chaudhary V et al (level V followed by level II)^11^,^16^ but inconsistent with Jha BC et al (level II followed by level III) and Mogre DA (level II followed by level V).^10^,^14^

Systemic features found in this study (63.07%) were similar to another Bangladeshi study.^13^ The clinical findings didn’t match with two similar regional studies from India and Pakistan where systemic symptoms were only 18% and 8% respectively.^10^,^11^ However Choudhury N et al found systemic symptoms in 36.36% of patients.^12^ Frontanilla JM et al found systemic symptoms more frequent in HIV-positive patients than HIV-negative patients(76% vs 12%).^15^ Khan R et al and Jha BC et al found FNAC positive in 90% and 85.7% cases respectively which were also similar with this study(83.9%).^8^, ^10^ On chest radiograph, Jha BC et al and Magsi PB et al found associated lung lesion in 16% and 7.5% cases respectively which were higher than ours (3.1%).^10^,^11^ But Choudhury N et al found associated lung lesion in 48.48% cases.^12^ In this study ESR was found raised in 70.76% cases. Jha BC et al found ESR raised in all but four patients.^10^ Magsi PB et al and Umer MF et al found ESR raised in 12.5% and 47.7% cases respectively.^11^,^17^

Anti-tuberculous chemotherapy is the mainstay in the management of TB lymphadenitis.^2^ The six months treatment may be sufficient for many patients, but it is difficult to define a clear cut ‘end point’ for assessing the efficacy of treatment of extra-pulmonary tuberculosis with delayed response.^2^ Surgery increase the cure rate with excellent cosmetic result and a low complication rate.^2^ Half of the patients (50.8%, 33) needed some form of surgical interventions. Jha BC *et al* treated 56 patients successfully with short course chemotherapy for six months where surgery was required rarely.^10^ A major limitation of this study was not performing culture for mycobacterium due to lack of facilities. However we depended mainly on clinical clues and other available investigations including FNAC.

## CONCLUSION

Cervical tuberculous lymphadenitis usually presents with unilateral, multiple, matted neck swelling in young adults. Female and low income group people affected more. FNAC could be a reliable tool for diagnosis. Anti-tuberculous chemotherapy is the mainstay of treatment. Surgical treatment is more useful in selected cases. Early diagnosis and treatment is critical in lowering the overall prevalence. Therefore, it is essential to have awareness regarding common presentations of CTL among the general populations as well as healthcare professionals working in the resource poor primary and secondary level hospitals.
